# Rare Brain Tumor in Infancy: Intraparenchymal Meningioma with Suspected Meningioangiomatosis in a Nine‑Month‑Old

**DOI:** 10.5334/jbsr.4125

**Published:** 2025-10-22

**Authors:** Kristian Jochems, Lukas Marcelis, Johannes Devos

**Affiliations:** 1 Department of Radiology, UZ Leuven, University Hospitals Leuven, 3000 Leuven, Belgium; 2 Department of Pathology, UZ Leuven, University Hospitals Leuven, 3000 Leuven, Belgium

**Keywords:** case report, pediatric, radiology, intraparenchymal meningioma, pediatric meningioma, meningioangiomatosis

## Abstract

An exceptionally rare case is described of intraparenchymal WHO grade 2 meningioma with suspected meningioangiomatosis in a nine‑month‑old boy presenting with absence seizures. MRI revealed a heterogeneously enhancing right frontal mass without dural attachment, encasing MCA branches. Histopathology confirmed atypical meningioma with adjacent perivascular meningothelial proliferation. Methylation profiling supported the diagnosis. Following gross total resection without adjuvant therapy, the patient remains seizure‑free with no recurrence at four‑year follow‑up.

*Teaching point:* Pediatric meningiomas are rare tumors that exhibit atypical presentations compared to their adult counterparts and should be included in the differential diagnosis of intra‑axial lesions in children.

## Introduction

Meningiomas represent one‑third of all CNS tumors but are uncommon in children (<2%) [1, 2]. While typically dural‑based, they may rarely be intraparenchymal, particularly in pediatric patients [3]. We describe a rare case of intraparenchymal meningioma with suspected meningioangiomatosis (MA) in an infant and highlight its clinical, histopathological, and imaging features.

## Case Report

**Figure 1 (A–B):**
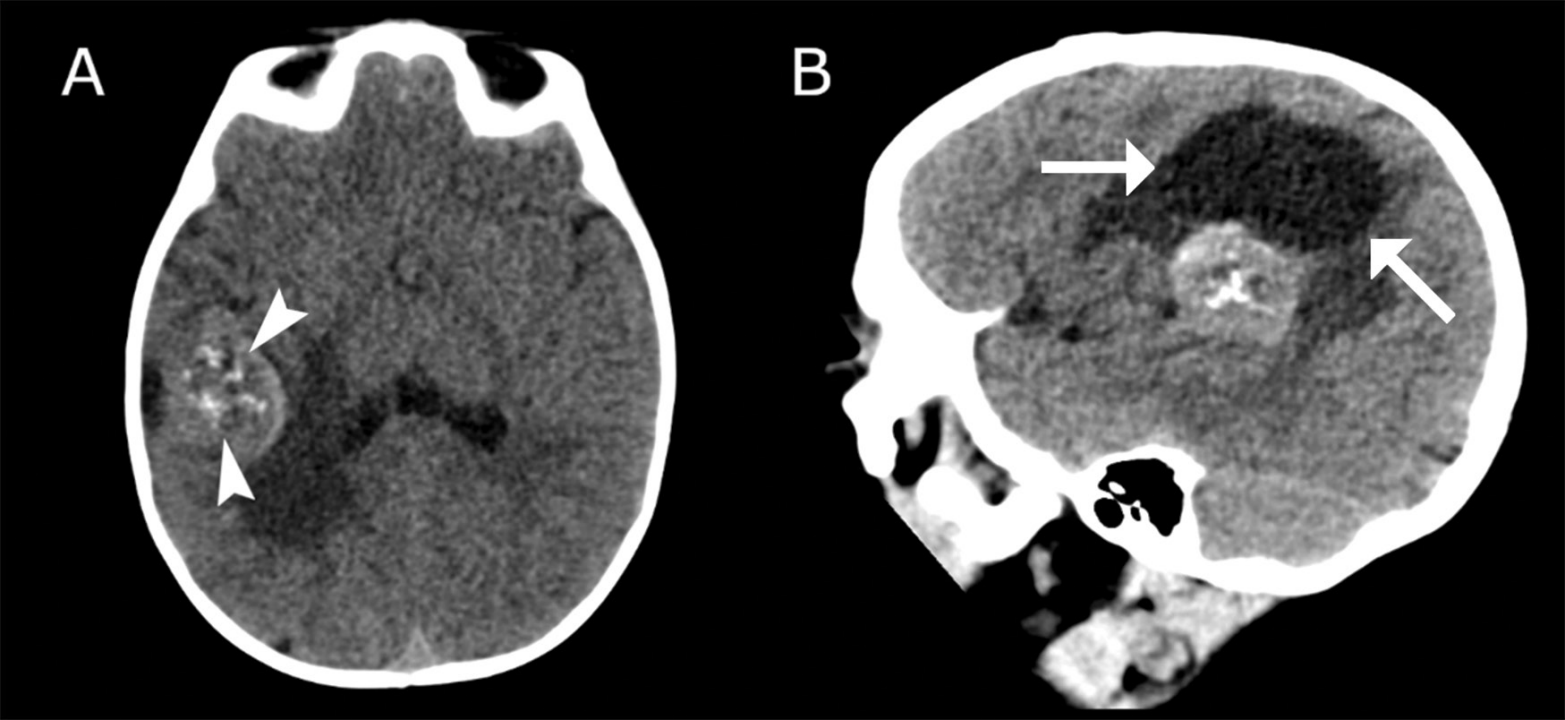
Imaging findings on non‑contrast CT brain. **(A)** Axial slices show a lesion in the right cerebral hemisphere with perilesional edema causing mass effect on the right lateral ventricle. Coarse calcifications are seen centrally within the lesion (arrowheads). **(B)** Sagittal slices show a large cystic component superiorly to and in close relationship with the lesion (arrows).

A nine‑month‑old boy presented with a one‑week history of daily absence episodes, without convulsions or loss of consciousness. Developmental history and family background were unremarkable. Neurological exam and EEG were normal. Unenhanced CT showed a solid, hyperdense right frontal mass with a hypodense center, coarse calcifications, and perilesional edema causing mass effect on the right lateral ventricle (Figure 1).

**Figure 2 (A–D):**
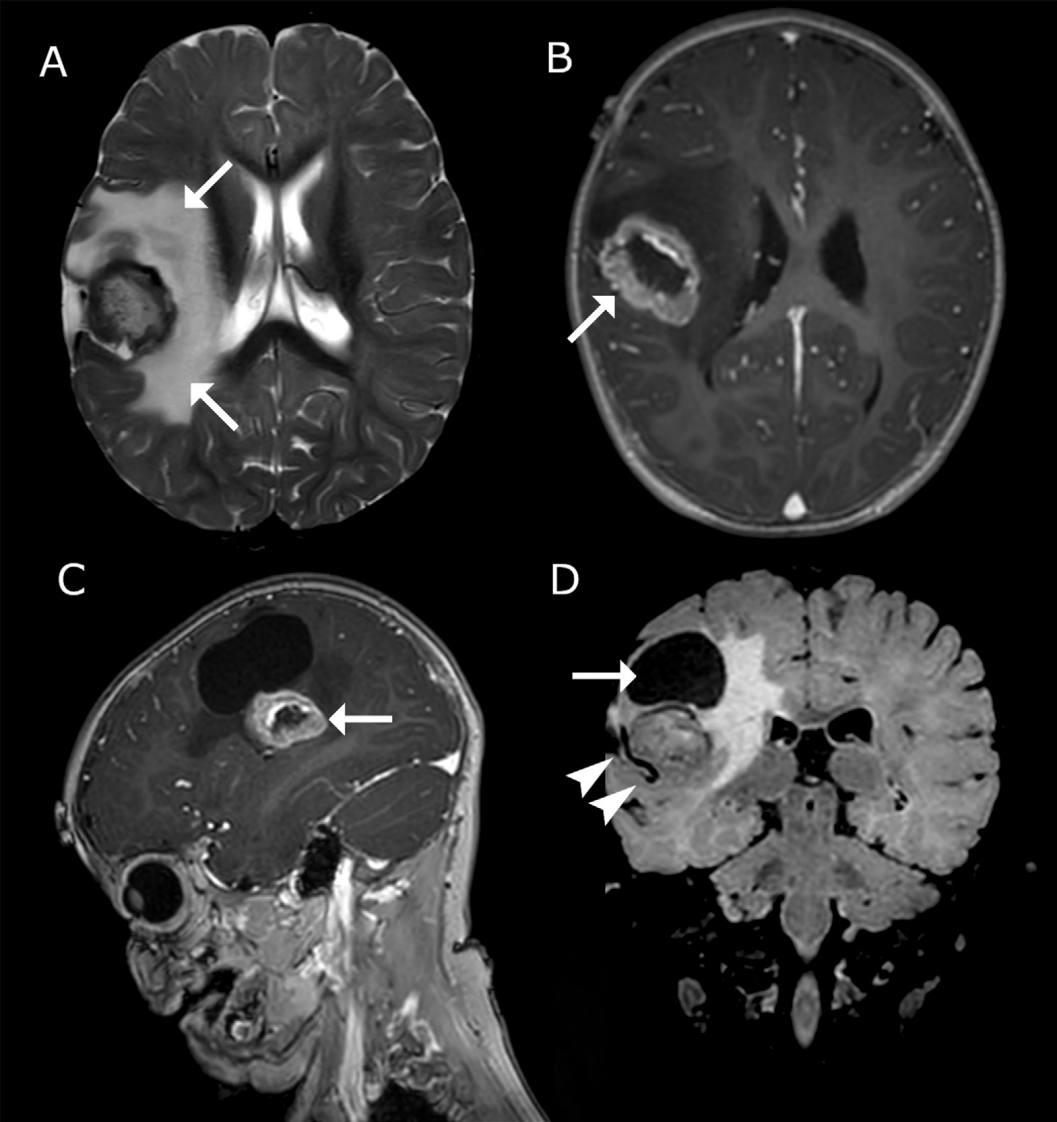
Imaging findings on 3‑Tesla MRI brain. **(A)** Axial T2‑wi showing a cortically based lesion in the right cerebral hemisphere with perilesional edema (arrows). **(B&C)** 3D T1‑wi; the peripheral, solid components show contrast‑enhancement (arrows). **(D)** FLAIR; coronal images demonstrate a large peritumoral cyst (arrow). In addition, a flow void of an MCA branch is seen traversing the lesion (arrowheads), in keeping with encasement.

On MRI, the solid component of the lesion enhanced heterogeneously, with encasement of branches of the middle cerebral artery (MCA; Figure 2) and with diffusion restriction. No dural connections were present.

The tumor was resected with the aid of neuronavigation. It was partially covered by cortex, and gross total resection was achieved, including the components encasing the MCA branches.

Histological evaluation identified two interconnected components: a central meningioma and adjacent perivascular meningothelial proliferations. The meningioma displayed classic whorls, cortical invasion, necrosis, and seven mitoses per 50 high‑power fields, qualifying for CNS WHO grade 2 under the 5th edition criteria.

Methylation profiling (Illumina EPIC array) analyzed via the DKFZ CNS classifier v12.5 classified the tumor as meningioma, subtype benign, subclass 3, with a score >0.90. Copy number profiling showed partial 11q loss.

**Figure 3 (A–D):**
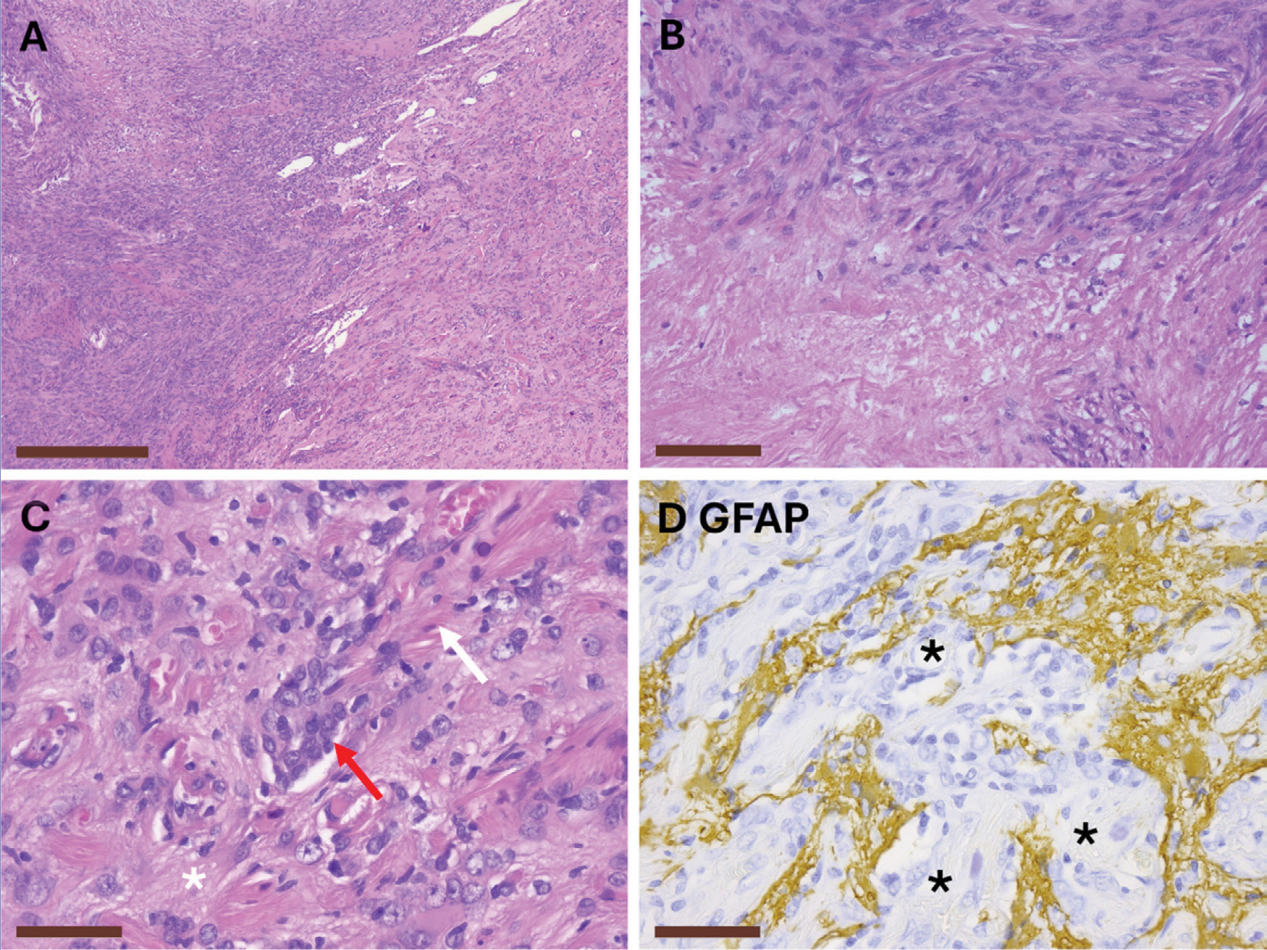
Histopathological findings of the resected specimen. **(A)** H&E overview of the meningioma bulk (left) and adjacent brain tissue (right), scalebar 500 µm; **(B)** H&E close‑up of the meningioma with necrosis (bottom), scalebar 100 µm; **(C)** H&E close‑up of the adjacent brain tissue, note the fine fibrillary glial tissue (asterisk), the fibrotic changes (white arrow), and the meningothelial cells often in close association with small vessels (red arrow), scalebar 50 µm; **(D)** GFAP immunohistochemistry stains the glial background with sparing of meningothelial cells in close relation with blood vessels (asterisk), scalebar 50 µm. All images are taken with a Leica DFC290 HD camera on a Leica DM2000 LED microscope.

Adjacent brain parenchyma exhibited fine fibrillary reactive gliosis, fibrosis, and meningothelial cells closely tracking small vessels, consistent with MA. GFAP immunohistochemistry highlighted the glial background while sparing the perivascular meningothelial elements, supporting the diagnosis (Figure 3).

The patient recovered without complications. No adjuvant therapy was administered. NF2 testing was negative. At four‑year follow‑up, MRI showed no recurrence, and the patient remained seizure‑free with normal development.

## Discussion

Although meningiomas are the most common CNS tumors in adults, pediatric cases are rare and differ significantly from adult presentations. They more frequently show atypical imaging findings, arise in unusual locations, and have more aggressive histological features [1, 2].

This case demonstrates the atypical nature of pediatric meningiomas through two key features. First, the intraparenchymal location is exceptionally rare, with only 52 cases reported globally [3]. Second, the tumor displayed dual histologic components: a WHO grade 2 meningioma accompanied by suspected MA. MA represents a rare cortical lesion involving fibro‑meningothelial cell proliferation around perivascular spaces [4], and this meningioma–MA combination has been described in 63 published cases [5]. Interestingly, these entities are increasingly recognized to constitute the same histogenetic spectrum, with potential implications for meningioma classification and prognostication [6–8].

The intraparenchymal location, cystic changes, absence of dural tail, and patient’s young age made meningioma an unlikely initial diagnosis. The differential included ependymoma, embryonal tumors, atypical teratoid/rhabdoid tumor, and high‑grade glioma. Imaging alone cannot reliably distinguish these entities, necessitating tissue diagnosis.

Atypical imaging features occur in approximately 15% of adult meningiomas but are more frequent in pediatric and intraparenchymal cases [1–3]. While such features typically correlate with higher grades and aggressive treatment in adults, pediatric meningiomas with atypical imaging generally maintain favorable prognoses despite more frequent WHO grade 2–3 histology [9]. Complete surgical resection remains the key prognostic factor [1, 2, 9]. Our patient achieved excellent outcomes following gross total resection, remaining seizure‑ and recurrence‑free with normal development at four‑year follow‑up.

## Conclusion

We report a unique case of a pediatric, intraparenchymal, WHO grade 2 meningioma with a suspected MA component. This case draws attention to the diagnostic challenges associated with atypical imaging and histological findings. Gross total resection led to an excellent outcome, with the patient remaining seizure‑ and recurrence‑free four years postoperatively. This case adds to the growing body of literature on pediatric meningiomas and supports ongoing efforts to refine their classification and prognostication—particularly in cases with overlapping pathology like MA.
